# Spatiotemporal variability in the structure of seagrass meadows and associated macrofaunal assemblages in southwest England (UK): Using citizen science to benchmark ecological pattern

**DOI:** 10.1002/ece3.5025

**Published:** 2019-03-04

**Authors:** Dan A. Smale, Graham Epstein, Mark Parry, Martin J. Attrill

**Affiliations:** ^1^ Marine Biological Association of the United Kingdom, The Laboratory Plymouth UK; ^2^ Ocean and Earth Science, National Oceanography Centre Southampton University of Southampton Southampton UK; ^3^ National Marine Aquarium Plymouth UK; ^4^ School of Biological and Marine Sciences University of Plymouth Plymouth UK

**Keywords:** coastal biodiversity, foundation species, monitoring, Seagrass beds, *Zostera marina*

## Abstract

Seagrass meadows underpin a variety of ecosystem services and are recognized as globally important habitats and a conservation priority. However, seagrass populations are currently impacted by a range of biotic and abiotic stressors, and many are in decline globally. As such, improved understanding of seagrass populations and their associated faunal assemblages is needed to better detect and predict changes in the structure and functioning of these key habitats. Here, we analyzed a large dataset—collected by recreational scuba divers volunteering on a citizen science project—to examine spatiotemporal patterns in ecological structure and to provide a robust and reliable baseline against which to detect future change. Seagrass (*Zostera marina*) shoot density and the abundance of associated faunal groups were quantified across 2 years at 19 sites nested within three locations in southwest UK, by collecting in situ quadrat samples (2,518 in total) during 328 dives. Seagrass shoot density and meadow fragmentation was comparable across locations but was highly variable among sites. Faunal abundance and assemblage structure varied between areas with or without seagrass shoots; this pattern was largely consistent between locations and years. Overall, increased seagrass density was related to increased faunal abundance and explained shifts in faunal assemblage structure, although individual faunal groups were affected differently. More broadly, our study shows that well‐funded and orchestrated citizen science projects can, to some extent, gather fundamental information needed to benchmark ecological structure in poorly studied nearshore marine habitats.

## 1 INTRODUCTION

Coastal vegetated habitats, such as seagrass meadows, kelp forests and salt marshes, have significant ecological and socioeconomic importance given that they underpin ecosystem services including habitat provision for fisheries species, biogenic storm defense, carbon capture, and storage and the maintenance of biodiversity (Barbier et al., 2011; Beaumont, Austen, Mangi, & Townsend, 2008; Bertocci, Araújo, Oliveira, & Sousa‐Pinto, 2015; Cullen‐Unsworth & Unsworth, 2013; Smale, Burrows, Moore, O'Connor, & Hawkins, 2013; Teagle, Hawkins, Moore, & Smale, 2017). However, in many regions, the persistence and integrity of these habitats, and the ecosystem services they provide, are threatened by a range of anthropogenic stressors, including fishing impacts, physical disturbance, invasive species, increased input of sediment, nutrients and pollutants, and ocean warming (Filbee‐Dexter & Wernberg, 2018; Kennish, 2001; Kirwan & Megonigal, 2013; Orth et al., 2006; Short & Wyllie‐Echeverria, 2009; Strain, Thomson, Micheli, Mancuso, & Airoldi, 2014). In order to document changes and impacts, and ultimately inform management and conservation strategies, robust baseline information on ecological structure and functioning is critical, yet for many systems, it remains inadequate often due to a lack of resources to undertake monitoring.

Seagrass meadows, in particular, are widely acknowledged for their ecological value as they play pivotal roles in natural carbon sequestration (Duarte, Kennedy, Marbà, & Hendriks, 2013; Fourqurean et al., 2012), sediment stabilization (Fonseca, 1989; van Katwijk, Bos, Hermus, & Suykerbuyk, 2010), and the provision of food and habitat for a myriad of associated organisms (Attrill, Strong, & Rowden, 2000; Bertelli & Unsworth, 2014; McCloskey & Unsworth, 2015; Nagelkerken et al., 2002). Extensive research from across the global distribution of seagrasses has shown that many populations have declined in recent decades, mostly due to decreased water quality (i.e., increased turbidity, nutrients, sediments), disease, and physical disturbance relating to coastal development and boating activities (Orth et al., 2006; Short et al., 2011; Waycott et al., 2009). In the United Kingdom, as elsewhere, seagrass meadows are a focal habitat for conservation but recent research has raised concerns over their ecological health (Jones & Unsworth, 2016) and the efficacy of monitoring and management (Jackson, Cousens, Bridger, Nancollas, & Sheehan, 2016).

Seagrass meadows in the United Kingdom comprise two species, *Zostera marina* L. and *Zostera (Zosterella) noltei* Hornemann, with the former being the more common species that can form extensive meadows in shallow subtidal sediments. These meadows are a focal habitat and protected feature of Marine Conservation Zones (MCZs) and are a named component of “Lagoons and Shallow Sandbanks” within the European Union Habitats Directive (Jackson et al., 2016). The inclusion of seagrass habitats within management frameworks is due in part to the belief that they support relatively high biodiversity compared to other habitats. Despite this statutory recognition, few studies have empirically examined variability in the structure of seagrass populations and their associated macrofaunal assemblages across multiple spatial or temporal scales in the United Kingdom (but see Jackson, Attrill, & Jones, 2006, Peters, McCloskey, Hinder, & Unsworth, 2015, Jones & Unsworth, 2016). Moreover, the lack of a co‐ordinated long‐term monitoring programme for many subtidal populations and habitats in the United Kingdom (but not all, for example see the Isles of Scilly; Bull & Keyton, 2016) has led to considerable uncertainties surrounding the health, structure, and long‐term trends of seagrass meadows (Jackson et al., 2016; Jones & Unsworth, 2016).

The aims of the current study were twofold. First, to conduct a robust assessment of the structure of seagrass populations and their associated macrofaunal assemblages across multiple spatial scales and years in SW England. It was envisaged that such a baseline could be used to detect ecological changes in coming years and decades. Second, to empirically examine relationships between seagrass population density and macrofaunal abundance and assemblage structure. Specifically, we examined: (a) whether faunal abundance/structure varies in areas with and without seagrass; (b) whether faunal abundance/structure is influenced by seagrass density and (c) whether these patterns are consistent in space and time. Crucially, we addressed these objectives by interrogating a large dataset generated by a citizen science project involving recreational scuba divers, allowing us to assess the usefulness of such initiatives for ecological monitoring.

## 2 MATERIALS AND METHODS

### 2.1 Project background

Data analyzed and presented here were collected as part of the Community Seagrass Initiative (CSI), a citizen science project led by the National Marine Aquarium, Plymouth UK, that aimed to increase education and awareness of the value of seagrass meadows in southwest England (for more information see http://www.csi-seagrass.co.uk). One component of the CSI project was to engage experienced recreational scuba divers by offering training and first‐hand experience of conducting scientific diving surveys. In total, between 2015 and 2017, >450 volunteer scuba divers completed training and survey dives at 22 sites. Before data collection commenced, divers received ~12 hr of training in survey techniques, identification, and enumeration of flora and fauna, and diving safety. The project was co‐ordinated by professional conservationists/ecologists and all divers had extensive experience of cold water (i.e., drysuit) diving. Even so, measures were taken to ensure that data included and analyzed here were robust, reliable, and repeatable. For the current study, we selected a subset of the entire survey effort and dataset for detailed analysis. First, all divers that participated in the project were categorized as either “competent” or “beginner” based on their individual skills and experience, and level of engagement with the project. For quality control, we removed all data collected by “beginners” prior to analysis (~31% of all data collected). Second, we only included data collected during the main “expedition” dives, which took place in summer (July to September) in both 2016 and 2017 (Table 1). These were periods of intense, regular fieldwork with a core team of well‐trained and highly experienced divers. Surveys that were conducted outside of these periods were predominantly training, reconnaissance or method development dives and were not included in the formal analysis. In total, the data used in the current study stemmed from 328 survey dives across 19 sites, which yielded 2,518 quadrat samples (see Table 1 and below).

**Table 1:** Summary and survey sites and timings and sampling effort for the current study

Location	Site #/name	Lat	Long	Survey periods	Total # dives	Total # quadrats
A. Plymouth and Looe	1. Cawsands	50.32857	−4.19582	Aug 16 and Aug 17	18	129
	2. Cellars	50.31037	−4.06538	Aug 16 and Sep 17	18	111
	3. Drake's Island	50.35773	−4.15345	Aug 16 and Aug 17	18	129
	4. Firestone	50.35992	−4.16088	Aug 16 and Aug 17	18	129
	5. Looe	50.35422	−4.43748	Aug 16 and Aug 17	18	129
	6. Ramscliffe	50.34178	−4.13015	Aug 16 and Aug 17	18	132
	7. Tomb Rock	50.30300	−4.07280	Aug 16 and Aug 17	18	135
B. Salcombe and Torbay	8. (Brixham) Breakwater	50.40062	−3.50383	Aug 16 and Aug 17	18	165
	9. Elberry Cove	50.40328	−3.54232	Aug 16 and Aug 17	20	195
	10. Fishcombe Cove	50.40302	−3.52232	Aug 16 and Aug 17	18	180
	11. Hope Cove	50.46498	−3.48780	Aug 16 and Aug 17	20	150
	12. Millstones Bay	50.45543	−3.52372	Aug 16 and Aug 17	20	150
	13. Salcombe	50.23270	−3.76937	Aug 16 and Sep 17	18	135
	14. Torre Abbey	50.45757	−3.53312	Aug 16 and Aug 17	18	135
C. Weymouth	15. PortlandHarbour West	50.59348	−2.45930	Aug 16 and Aug 17	16	120
	16. Ringstead Bay	50.63043	−2.35427	Aug 16 and Aug 17	12	87
	17. Weymouth Bay Middle	50.62452	−2.43167	Aug 16 and July 17	14	104
	18. Weymouth Bay West	50.62157	−2.43748	July 16 and Aug 17	18	135
	19. Weymouth Pier	50.60977	−2.44490	Sep 16 and Aug 17	10	68

### 2.2 Study region

**Figure 1:**
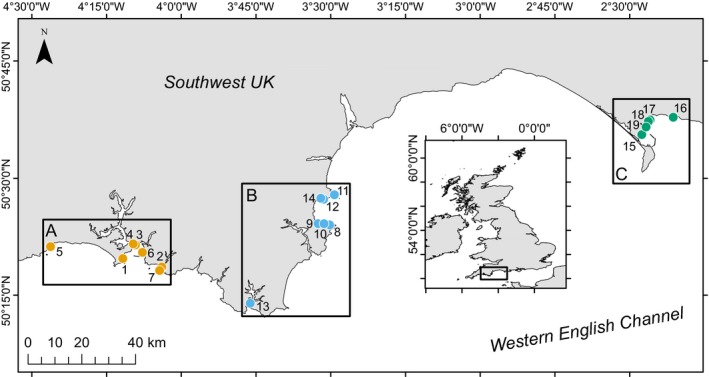
Study sites across three regions in the Southwest UK (location of study region within the UK shown by box on inset map). Location A = orange, location B = blue, location C = green

Surveys were conducted along a ~250 km stretch of coastline in southwest England (UK), spanning parts of Cornwall, Devon and Dorset (Figure 1). Three main locations were selected a priori based on known existence of seagrass populations and presence of extensive suitable habitat in terms of depth and substrate type (i.e., sediment rather than reef); Plymouth and Looe (location “A”), Salcombe and Torbay (B), and Weymouth (C). Within each location, 5–7 sites were selected (at random from a larger possible pool) for survey (Table 1). Sites were selected based on known presence of seagrass meadows (e.g., from NBN records, or existing habitat/biotope maps), and the presence of seagrass habitat was visually confirmed during presurvey training dives. Survey sites were situated in ~1–5 m depth (below chart datum). All locations include areas specifically managed for their conservation value (i.e., Plymouth Sound SAC, Salcombe Estuary SSSI, Studland to Portland SAC, Torbay MCZ, Lyme Bay, and Torbay SAC).

### 2.3 Survey design and data collection

The structure of seagrass habitats was quantified by haphazardly placing quadrats (0.25 m^2^) at each site. Within each quadrat, divers enumerated the number of seagrass shoots (*Zostera marina*) and the abundance of coarse faunal groups. To avoid issues relating to misidentification and the need for comprehensive training, surveys were conducted at a coarse taxonomic level. While this “simplification” in determining ecological pattern did not allow for a robust quantification of biodiversity (i.e., species richness), it did allow for many samples to be collected across large spatial scales with a high degree of confidence, in order to assess broad ecological patterns. The nine faunal groups were sponges, ascidians, cnidarians, bryozoans, crustaceans, echinoderms, worms, molluscs, and fish. Epifauna found on both seagrass and the underlying substratum were recorded, whereas infauna and small and/or highly mobile species or organisms (i.e., amphipods, juvenile crabs/fish) were not recorded. For colonial organisms, each colony (rather than individual) was counted. All quadrats were photographed in situ and any uncertainties with faunal identification were subsequently addressed. Due to logistics relating to weather, site characteristics, and organizing volunteer dive teams, the sampling effort varied between sites, ranging from 68 quadrats to 195 quadrats and averaging 132 ± 29 quadrats (Table 1).

### 2.4 Data analysis

To assess differences in the composition of seagrass habitats, the associated fauna and their interactions, a variety of generalized linear mixed models (GLMMs) and permutational analyses of variance (PERMANOVAs) tests were constructed on univariate and multivariate responses, respectively. In all cases, models were initially constructed on the entire survey dataset and comparisons were made between samples either with or without seagrass (i.e., shoot density “=0” vs. “>0”). Secondly, only those quadrats which contained seagrass (i.e., shoot density “>0”) were selected for further analysis to examine within‐seagrass effects. In all cases, inferences were made at the “location” level (categorical; 3 levels), with “site” (categorical; 19 levels) included as a random factor.

For seagrass meadow composition analysis, the probability of finding seagrass in a single quadrat (used as a proxy of bed fragmentation) was analyzed using presence–absence data and a binomial GLMM (bGLMM), while number of seagrass shoots per quadrat was analyzed using a negative binomial GLMM (nbGLMM). In both cases, the response was modeled as a function of “year” (categorical; 2 levels) and “location.” Differences in total faunal abundance between areas of seagrass presence and absence were assessed using a nbGLMM with “year,” “location,” and “seagrass presence–absence” (categorical; 2 levels) as fixed factors; and, where seagrass was present, total faunal abundance was modeled as a function of “year,” “location,” and “seagrass shoot abundance” (discrete; log transformed due to right skewed errors). “Year” was treated as a fixed factor as our a priori expectation was that spatial variability patterns would be consistent between years (i.e., no effect of “year”) and because with only two levels of the factor, it was not possible to generalize across the wider “random” effect of year. The abundance of four dominant faunal groups (Supporting Information Table S1) were also modelled as individual univariate responses and fitted to the same models as total faunal abundance. The multivariate response of all 9 faunal groups recorded in each quadrat was also compared between areas of seagrass presence and absence with PERMANOVA using the same model construction as total faunal abundance. Where seagrass was present, the multivariate response was modeled as a function of “year,” “location,” and “seagrass density category” (categorical; 4 levels). Density category was used instead of shoot abundance to better investigate and display the influence of seagrass density on associated fauna in a multivariate context. Density categories were A = absent (not included in analysis), B = 1–5, C = 6–10, D = 11–18, E = >18 seagrass shoots per 0.25 m^2^.

For every model, all fixed‐factor interactions were investigated including three‐way combinations. All multivariate analyses were conducted on fourth‐root transformed data (to downweight the influence of dominant faunal groups) and Bray–Curtis similarity matrices. bGLMMs used complementary log–log link functions, while nbGLMMs used log links. Validation of all GLMMs was carried out using diagnostic quantile–quantile plots and predicted versus residual plots, both using simulated scaled residuals; while significance of each model coefficient was assessed using likelihood ratio tests. Where significant effects of categorical fixed factors were identified pairwise differences between levels of factors were tested using post hoc Chi‐squared tests with Holm adjusted *p*‐values for GLMMs, and pairwise PERMANOVA for multivariate data. The scale and direction of effects from significant discrete fixed factors were visualized graphically; multivariate effects were visualized using threshold metric multidimensional scaling (tmMDS) on bootstrap averages with their 95% confidence regions.

All univariate statistics were carried out in R 3.5.1 (R Core Team, 2018), while multivariate statistics were conducted in PRIMER‐e version 7 (Clarke, Gorley, Somerfield, & Warwick, 2014). bGLMMs and nbGLMMs were fitted using the *glmer *and *glmer.nb *commands, respectively, both from the *lme4 *package (Bates, Machler, Bolker, & Walker, 2015). Model validation plots were created using the *DHARMa* package (Hartig, 2018); while univariate pairwise tests were conducted using the *testInteractions* command from the *phia* package (De Rosario‐Martinez, 2015). All PERMANOVAs were run with 1,999 permutations of residuals under a reduced model with Type 3 (partial) sums of squares. tmMDS plots were visualized using 50 restarts and a minimum stress of 0.01. Bootstrap averages were calculated with 75 bootstraps per group, with automatic selection of dimensions based on *ρ* > 0.99. Data manipulation were carried out using *dplyr* (Wickham & Francois, 2015), graphs were created using *ggplot2* (Wickham, 2009) or PRIMER, and mapping (Figure 1) was carried out within ArcMap 10.3.1. Where relevant all data are shown ±standard error.

## 3 RESULTS

### 3.1 Seagrass meadow structure

**Figure 2:**
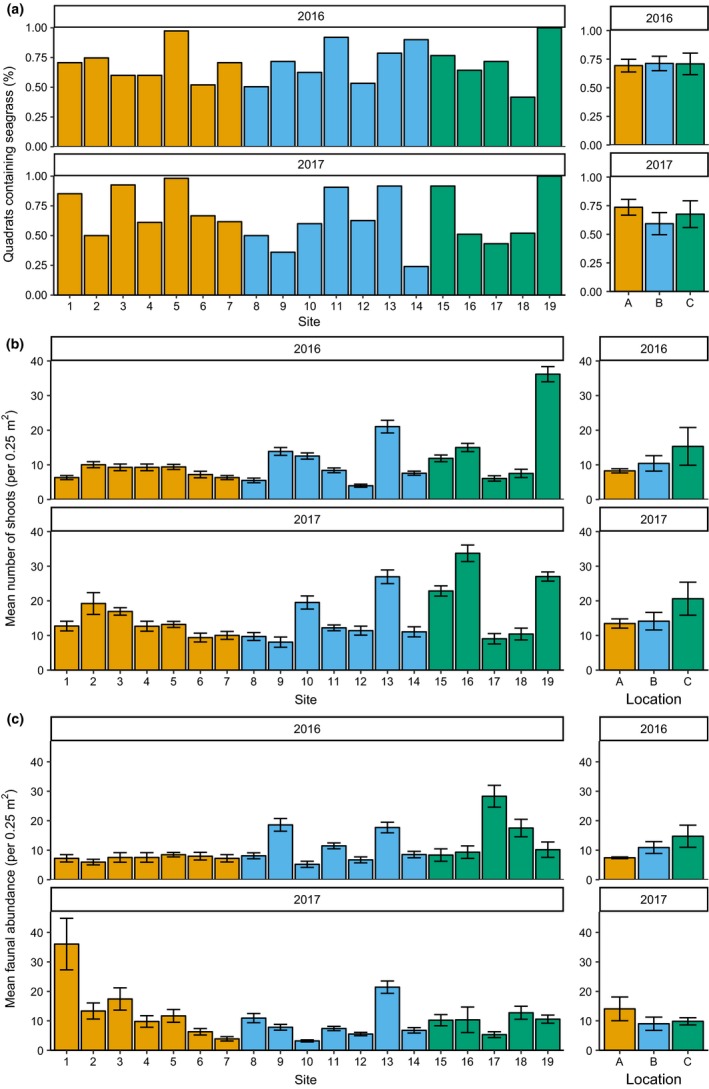
Seagrass meadow fragmentation, shoot density, and faunal abundance for each survey site (±quadrat *SE*) and location (±site *SE*) in each year. (a) Percentage of quadrats containing seagrass was used as a proxy for bed fragmentation, where an increase in percentage would indicate lower fragmentation. (b) Mean number of shoots (per 0.25 m^2^) was calculated within areas of seagrass only, as was mean faunal abundance (per 0.25 m^2^) (c)

Seagrass populations were widely distributed across the study region, with 68% of all samples returning seagrass presence. All three survey locations were dominated by seagrass with similar levels of bed fragmentation (quadrats containing seagrass: Plymouth and Looe, “A” = 71.5 ± 4.3%, Salcombe and Torbay, “B” = 65.3 ± 5.8%, Weymouth, “C” = 69.2 ± 7.1%; Figure 2). At the site level, lowest fragmentation and highest shoot density were recorded at site 19 in location C where all quadrats contained seagrass and mean shoot density was 36.2 ± 2.2 shoots per 0.25 m^2 ^in 2016. Highest site level fragmentation and lowest shoot density were recorded in location B with only 24.0% of quadrats containing seagrass at site 14 in 2017 and a shoot density of 3.9 ± 0.4 per 0.25 m^2 ^recorded at site 12 in 2016 (Figure 2). Considering all sites together, there were no significant differences in meadow fragmentation between locations in either year, and only location B had a significant difference in fragmentation between years, with an increase in fragmentation in 2017 (Table 2, Supporting Information Table S2). There was also no significant effect of location on seagrass shoot density (Table 2); however, in both survey years location C had highest mean shoot density (15.3 ± 5.4 and 20.6 ± 4.8 shoots per 0.25 m^2 ^in 2016 and 2017, respectively), and location A had the lowest mean density (8.3 ± 0.6 and 13.5 ± 1.3 shoots per 0.25 m^2^; Figure 2). There was a significant difference in shoot density between years, with all locations increasing from 2016 to 2017 (Table 2, Supporting Information Table S2). For both meadow fragmentation and shoot density, there was high site‐level variation (Figure 2, Table 2).

**Table 2:** GLMMs for two seagrass bed composition metrics, identifying the effect of year and location

Coefficient	Grps	Seagrass present–absent			Number of shoots (per 0.25 m^2^)		
Random		Stdev	*χ* ^2^	*p*	Stdev	*χ* ^2^	*p*
Site	19	0.52	223.94	**<0.001**	0.38	359.09	**<0.001**

Fixed	*df*	MS	*χ* ^2^	*p*	MS	*χ* ^2^	*p*
Year	1	1.70	1.74	0.188	140.32	132.98	**<0.001**
Location	2	0.32	0.65	0.724	1.38	2.56	0.278
Year*Location	2	8.09	15.81	**<0.001**	1.31	2.56	0.278

Note

Seagrass presence–absence data was used as a proxy for bed fragmentation and was analyzed using a bGLMM, while seagrass density (number of shoots per 0.25 m^2^) was analyzed using a nbGLMM. Each coefficient is shown with the number of groups (Grps) or degrees of freedom (*df*), along with the associated standard deviation (Stdev), mean‐squares (MS), chi‐squared value (*χ* ^2^), and *p*‐value (*p*). Significant coefficients shown in bold (*ɑ* < 0.05).

### 3.2 Abundance of associated fauna

**Figure 3:**
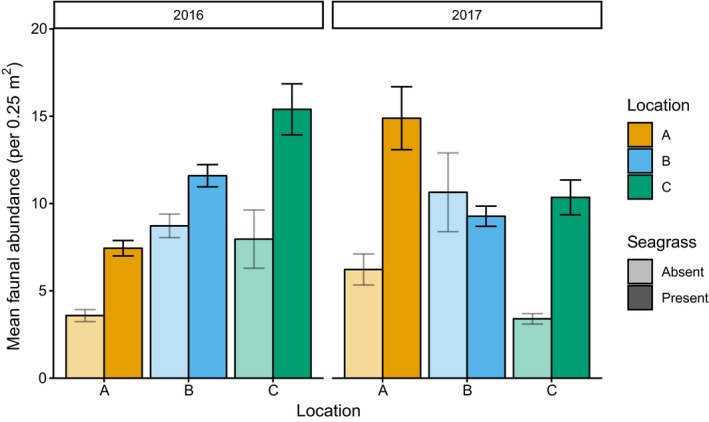
Mean faunal abundance (±*SE*) in each location and year for quadrats containing seagrass (darker bars to the right) and lacking seagrass (lighter bars to the left)

The presence of seagrass significantly affected mean faunal abundance within each year and location; however, the magnitude and direction of the effect was not consistent, as highlighted by a significant three‐way interaction (Table 3, Supporting Information Table S3). In all but one contrast, the presence of seagrass was related to a significant increase in mean faunal abundance with an average increase of 115.6 ± 28.2% when compared to areas lacking seagrass (Figure 3). The exception was in 2017 at location B where there was a significant reduction in mean faunal abundance where seagrass was present; however, this change was relatively small, from 10.6 ± 2.3 to 9.3 ± 0.6 individuals per 0.25 m^2 ^(Figure 3).

**Table 3:** nbGLMMs of total faunal abundance, comparing quadrats containing and lacking seagrass, and modeling the effect of the number of shoots in quadrats containing seagrass

Coefficient	Total faunal abundance						
	Seagrass presence–absence				Number of shoots (per 0.25 m^2^)		
Random	Grps	Stdev	*χ* ^2^	*p*	Stdev	*χ* ^2^	*p*
Site	19	0.30	142.65	**<0.001**	0.40	157.27	**<0.001**

Fixed	*df*	MS	*χ* ^2^	*p*	MS	*χ* ^2^	*p*
Year	1	1.17	2.55	0.110	1.61	1.65	0.199
Location	2	1.03	2.21	0.331	0.64	0.31	0.859
Seagrass	1	85.67	72.72	**<0.001**	126.24	111.43	**<0.001**
Year*Location	2	40.19	75.57	**<0.001**	20.65	24.57	**<0.001**
Year*Seagrass	1	1.09	1.01	0.314	0.02	0.17	0.682
Location*Seagrass	2	33.45	60.27	**<0.001**	14.57	29.55	**<0.001**
Year*Location*Seagrass	2	9.24	17.10	**<0.001**	3.36	6.60	**0.037**

The effect of year and location was also considered in each model. The coefficent “Seagrass” refers to a binary predictor in the seagrass presence–absence model, and a discrete predictor in the number of shoots model. Each coefficient is shown with the number of groups (Grps) or degrees of freedom (*df*), along with the associated standard deviation (Stdev), mean‐squares (MS), chi‐squared value (*χ* ^2^), and *p*‐value (*p*). Significant coefficients shown in bold (*ɑ* < 0.05).

When considering the four dominant faunal groups individually, all were significantly affected by seagrass presence–absence (Supporting Information Table S5) However, the direction of effects was only consistent for cnidarians and bryozoans (Supporting Information Figure S1), which increased in abundance in areas of seagrass for every within year–location contrast and this change was significantly different in all but one instance for both groups (Supporting Information Table S6). In contrast, molluscs and worms did not show a consistent pattern between areas of seagrass presence–absence (Supporting Information Figure S1) and had few significant pairwise effects within year–location contrasts (Supporting Information Table S6).

Considering only samples containing seagrass, the highest mean faunal abundance was recorded in location A at site 1 with 36.0 ± 8.7 individuals per 0.25 m^2 ^in 2017 and lowest in location B at site 10 with 3.2 ± 0.4 individuals per 0.25 m^2 ^in 2016 (Figure 2). However, considering all sites together, there were no significant differences in mean faunal abundance between locations in either year (Table 3, Supporting Information Table S4). However, faunal abundance was significantly different between years in each location, although the magnitude and direction of change were not consistent as highlighted by a significant “year*location” interaction (Figure 2, Table 3). In 2016, location C had the highest mean faunal abundance (14.7 ± 3.8 individuals per 0.25 m^2^) and location A had the lowest (7.4 ± 0.3 individuals per 0.25 m^2^); however, in 2017, this pattern was reversed with location A having highest faunal abundance (14.1 ± 4.0 per 0.25 m^2^) and locations B and C having lowest abundance (B = 9.0 ± 2.3; C = 9.8 ± 1.2 per 0.25 m^2^).

**Figure 4:**
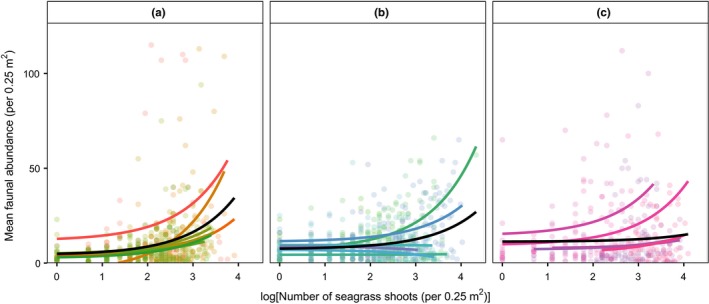
Scatter plots indicating the effect of seagrass density on mean faunal abundance within each location separately. Each point represents a single quadrat, with colors separating individual sites (A and B number of sites = 7, C = 5). Colored lines are exponential smoothing functions for each site, while black lines are exponential smoothing functions for each location based on fitted values from the global nbGLMM

Seagrass shoot density was significantly related to total faunal abundance, although the magnitude of the effect differed between locations (Table 3; Figure 4). For all three locations, an increase in the number of seagrass shoots was related to an increase in total faunal abundance, with the effect being largest in location A and smallest in location C (Figure 4). When considering the four dominant groups individually, only cnidarian and bryozoan abundance was significantly related to seagrass shoot density (Supporting Information Table S7, Figure S2). In all cases, an increase in shoot density was related to an increase in abundance; however, as found for total abundance, the magnitude of the effect differed between locations. In general, shoot densities in locations A and B had the largest effect on cnidarian and bryozoan abundances (Supporting Information Figure S2).

### 3.3 Multivariate structure of associated fauna

**Figure 5:**
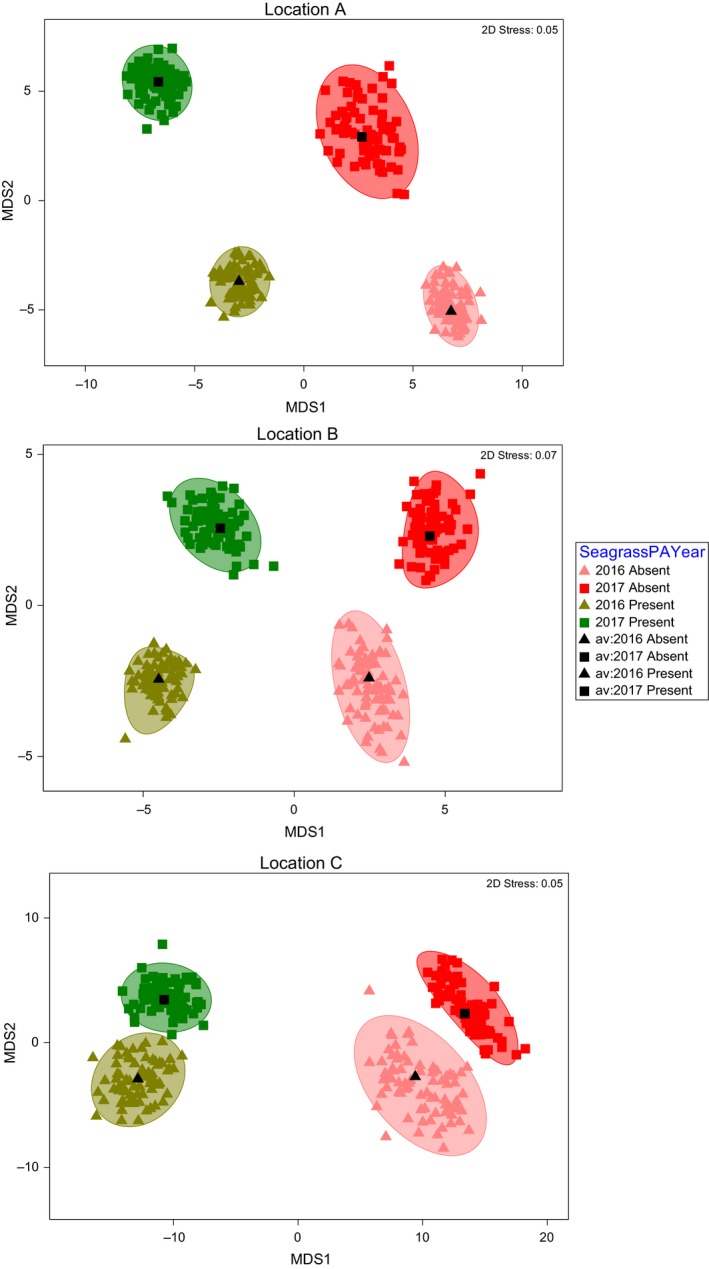
Threshold metric multidimensional scaling (tmMDS) plots of bootstrapped average fauna community data from all quadrats sampled for each location. Each tmMDS is grouped by seagrass presence absence and year. Bootstrapping and tmMDS based on Bray–Curtis distance matrices constructed from 4th‐root transformed data

**Figure 6:**
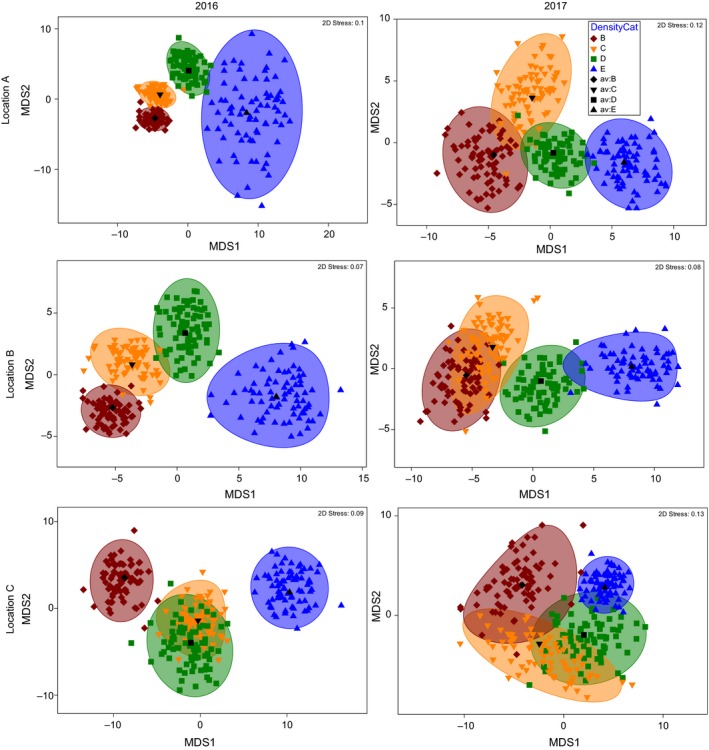
Threshold metric multidimensional scaling (tmMDS) plots of bootstrapped average fauna community data from quadrats containing seagrass for each location and year. Each tmMDS is grouped by seagrass density category. Bootstrapping and tmMDS based on Bray–Curtis distance matrices constructed from 4th‐root transformed data.

There was a significant difference in faunal assemblages between areas containing and lacking seagrass, although the magnitude of the effect was not consistent, as highlighted by a significant three‐way interaction in the multivariate PERMANOVA (Table 4). When visualizing the multivariate data the direction of the effect was consistent between locations and years (Figure 5), and for every pairwise contrast within each location and year, there was a significant difference in faunal assemblage structure between seagrass presence–absence (Supporting Information Table S8). In areas containing seagrass, there was some disparity in faunal assemblage structure between locations and years (Supporting Information Figure S3), although the only significant pairwise dissimilarity was recorded between locations A and B in 2016 (Supporting Information Table S9). There was, however, a clear significant effect of seagrass density category on the faunal community, although the magnitude of effect differed within each location and year (Table 4). In every location and year, seagrass density category had a similar direction of effect on the faunal community, with clustering from lowest to highest density category across the *X*‐axis of bootstrapped multivariate means (Figure 6). There were clear differences in the magnitude of the effect, with many overlapping clusters, and least defined clusters in location C in 2017 (Figure 6).

**Table 4:** PERMANOVAs of the multivariate abundance of nine seagrass associated faunal groups, comparing quadrats containing and lacking seagrass, and modeling the effect of seagrass density category in quadrats containing seagrass

Coefficient	Multivariate faunal abundance							
	Seagrass presence–absence				Seagrass density category			
	*df*	MS	*F*	*p*	*df*	MS	*F*	*p*
*Random*								
Site	16	15,953	21.22	**<0.001**	16	12,965	16.32	**<0.001**
*Fixed*								
Year	1	19,091	25.43	**<0.001**	1	20,522	14.03	**<0.001**
Location	2	22,747	1.75	0.057	2	17,034	1.27	0.258
Seagrass	1	41,731	55.58	**<0.001**	3	11,857	6.27	**<0.001**
Year*Location	2	7,090	9.44	**<0.001**	2	5,383	6.78	**<0.001**
Year*Seagrass	1	1,204	1.60	0.198	3	1,797	2.26	**0.016**
Location*Seagrass	2	10,177	13.55	**<0.001**	6	1,824	2.30	**0.002**
Year*Location*Seagrass	2	1,669	2.22	**0.034**	6	1,431	1.80	**0.019**

Note

The effect of year and location was also considered in each model. The coefficient “Seagrass” refers to a binary predictor in the seagrass presence–absence model, and a categorical predictor with four levels in the seagrass density category model. Each coefficient is shown with the associated degrees of freedom (*df*), mean‐squares (MS), *F*‐value (*F*), and *p*‐value (*p*). Significant coefficients shown in bold (*ɑ* < 0.05).

## 4 DISCUSSION

We recorded extensive seagrass‐dominated habitat across the study region, although seagrass population density and the structure of associated faunal assemblages varied across multiple spatial scales. For example, seagrass fragmentation and shoot density values were comparable between the three locations but highly variable between sites. High variability in shoot density between sites situated a few kilometers apart is a frequently observed pattern in seagrass‐dominated regions (Barberá et al., 2005; Fourqurean, Willsie, Rose, & Rutten, 2001; Leriche et al., 2006; Middelboe, Sand‐Jensen, & Krause‐Jensen, 2003), and here, we recorded a fourfold difference in mean shoot density between sites situated <10 km apart. In the absence of multiparameter environmental data and manipulative experiments, it is not possible to determine the underlying drivers of such variability. That said, by drawing on previous research, it is possible to cautiously infer likely mechanisms driving observed variability at this spatial scale. Sites within locations were positioned at varying levels of wave exposure (although all were very‐to‐moderately sheltered from wave action) and depth, and varying distances to geomorphological features (e.g., headlands), riverine inputs and intense coastal development. As such, it is highly likely that key environmental variables, including light availability (due to differences in both depth and turbidity), sedimentation rates, water quality, and physical disturbance from both wave action and anchoring, differed between sites. These variables are known to affect shoot density and habitat fragmentation (Cabaço, Machás, Vieira, & Santos, 2008; de Boer, 2007; Francour, Ganteaume, & Poulain, 1999; Frederiksen, Krause‐Jensen, Holmer, & Laursen, 2004; Middelboe et al., 2003; Unsworth, Williams, Jones, & Cullen‐Unsworth, 2017; West, 1990) and are likely to be important at this spatial scale in the southwest UK.

We also detected high variability at the smallest spatial scale between quadrats positioned meters apart. Pronounced variability in the structure of seagrass meadows at small spatial scales has been reported previously (Balestri, Cinelli, & Lardicci, 2003; Barberá et al., 2005) and is a characteristic feature of nearshore marine habitats in general (Fraschetti, Terlizzi, & Benedetti‐Cecchi, 2005; Smale, Kendrick, & Wernberg, 2010). Drivers of such variability include sporadic grazing pressure (Tomas, Turon, & Romero, 2005), physical disturbance (Milazzo, Badalamenti, Ceccherelli, & Chemello, 2004), bioturbation (Edward & Mark, 1998) and differences in microhabitat structure (Balestri et al., 2003). Conversely, we observed relatively minimal variability between locations, situated ~40 to ~140 km apart, suggesting that these distinct locations support a similar diversity of habitats and seagrass populations. Indeed, as all locations were dominated by the same species (*Zostera marina*) and likely encompassed a similar range of habitats in terms of wave exposure, depth, water quality and proximity to estuaries and human impact, it is perhaps unsurprising that variability at this scale was relatively unimportant. Similar patterns have been observed in seagrass‐dominated systems elsewhere (Barberá et al., 2005).

The structure of seagrass meadows did vary somewhat between the survey years, as fragmentation and shoot density differed between 2016 and 2017 at one and all of the three locations, respectively. With only 2 years of survey data, it is not possible to discern long‐term temporal trends or infer drivers of this pattern, but it is worth noting that pronounced interannual variability in seagrass population density has been observed previously (Bull & Keyton, 2016; Frederiksen et al., 2004; Saunders et al., 2015), as a consequence of interannual variability in wave disturbance (Frederiksen et al., 2004), light and nutrient availability (Moore, Shields, & Parrish, 2014; Saunders et al., 2015) or climatic variables (Marbà & Duarte, 1997), for example. Such between‐year variability has important implications for monitoring programmes and condition assessments, as high‐resolution sampling is needed in order to differentiate between “noise” and “trends.” This is certainly not the case for many UK seagrass populations, which are routinely monitored every ~6 years (see Jackson et al., 2016 for further discussion).

Faunal abundance and assemblage structure varied between areas with and without seagrass, with largely consistent patterns across years and locations. Specifically, in five of the six year/location combinations, faunal abundance was greater in quadrats containing seagrass compared with those without, and for all year/location combinations multivariate faunal assemblage structure differed with seagrass presence/absence. This strongly suggests that the occurrence of seagrass influences associated faunal assemblages and elevates faunal abundance (and presumably secondary productivity) compared with unvegetated sediments. While this pattern is largely intuitive, it has rarely been demonstrated across multiple spatiotemporal scales and, to our knowledge, this is this first empirical demonstration of such a pattern across ~100 s km of *Zostera* dominated habitat in the northeast Atlantic. Previous work at smaller scales has also shown that faunal assemblages within‐seagrass meadows are distinct and generally more diverse and abundant compared with those found in unvegetated sediments (Boström & Bonsdorff, 1997; Ferrell & Bell, 1991; Fonseca, Hutchings, & Gallucci, 2011; Fredriksen, Backer, Boström, & Christie, 2010; Hirst & Attrill, 2008; McCloskey & Unsworth, 2015; Mills & Berkenbusch, 2009; Turner & Kendall, 1999). Variability in faunal community structure may be due to the fact that seagrass offers increased structural complexity (Gartner, Tuya, Lavery, & McMahon, 2013; Webster, Rowden, & Attrill, 1998) and habitat area (Attrill et al., 2000), refuge from predation and disturbance (Peterson, 1982), elevated food availability (Vizzini, Sarà, Michener, & Mazzola, 2002), altered sediment characteristics (Frost, Rowden, & Attrill, 1999), and increased retention of particles, such as propagules and particulate organic matter (Christoffer & Erik, 2000; Thorsten, 1998). In the current study, we recorded consistently higher abundances of sessile taxa (e.g., cnidarians, bryozoans) in areas containing seagrass, most likely as seagrass populations offer biogenic substrate for settlement and growth of these organisms (Balata, Nesti, Piazzi, & Cinelli, 2007; Demers, Knott, & Davis, 2015; Mabrouk, Ben Brahim, Hamza, & Bradai, 2015).

At all locations, we recorded significant positive relationships between shoot density and faunal abundance, although the strength of relationships was highly variable. We also observed significant shifts in multivariate faunal assemblage structure along a gradient of shoot density, despite the coarse level of taxonomy applied. Given that these patterns were relatively consistent in both space and time, our results strongly suggest that seagrass populations facilitate associated macrofauna in this region. Again, while this pattern has been described previously (Attrill et al., 2000; Bell & Westoby, 1986b; McCloskey & Unsworth, 2015), our study has demonstrated this relationship holds across multiple spatial scales, providing robust evidence for the importance of seagrass habitats in structuring faunal populations and the wider benthic community. Intuitively, increased shoot density will likely result in a greater habitat area available for sessile organisms, while dense meadows may also trap and retain larvae and thereby increase settlement and recruitment rates (Christoffer & Erik, 2000; Thorsten, 1998). Additionally, increased shoot density has been linked with higher abundances of both infauna (Fonseca et al., 2011; Gambi, Conti, & Bremec, 1998) and small epifauna (Bell & Westoby, 1986a; Boström & Bonsdorff, 1997), which may serve as prey for the larger mobile faunal groups recorded here (e.g., crustaceans, echinoderms, fish). Furthermore, increased shoot density may also lead to greater trapping and retention of detritus and particulate organic matter (originating from both within and outside the seagrass meadow), thereby increasing food supply, sustaining local food webs and promoting faunal abundances (Lee, Fong, & Wu, 2001; Samper‐Villarreal, Lovelock, Saunders, Roelfsema, & Mumby, 2016). Increasing shoot density has also been to shown to promote the richness and abundance of infaunal assemblages (Webster et al., 1998). While the mechanisms underpinning facilitation remain unclear, the importance of seagrass as habitat for associated macrofauna is unequivocal.

The survey data presented and analyzed in this study were collected by volunteers as part of a citizen science project. The number of citizen science activities has increased rapidly in recent years, in line with technological advances (e.g., social media and data sharing platforms, tailored apps, etc.) and wider awareness of environmental issues and such projects are increasingly important for conservation (Jones, Unsworth, McKenzie, Yoshida, & Cullen‐Unsworth, 2018). Concurrently, the resources allocated to national and regional governments are in many cases insufficient to achieve effective monitoring (Rush & Solandt, 2017), and fundamental ecological information is lacking for many marine populations, habitats, and locations (Jackson et al., 2016; Smale et al., 2013). As such, it has been suggested that citizen science projects can be used to collect data to support conservation and management (Stuart‐Smith et al., 2017), although any information collected and used in this manner must be scientifically robust and reliable (Hyder, Townhill, Anderson, Delany, & Pinnegar, 2015). Here, the benefits of the citizen science approach were threefold. First, using volunteer recreational divers to conduct surveys was highly cost‐effective, as no funding was needed to cover salaries, and the total cost was considerably lower than contracting commercial scientific divers to complete the work. This, in effect, allowed for greater spatial and temporal coverage of the surveys than would have otherwise been possible. Second, the citizen science approach combined with a targeted advertising campaign and use of social media meant that a large number of volunteers were recruited and trained. This resulted in higher availability of trained divers than would likely have occurred within an academic or government agency setting. Third, the dive surveys were one component of the larger CSI project, which had the broader aim of increasing awareness of the importance of, and threats to, seagrass meadows in the region. The diving surveys were high profile and attracted public attention, feeding into a wider education and outreach programme. The level of public engagement would have perhaps been lower in an academic or government agency setting.

On the other hand, there were several limitations associated with the citizen science approach that need to be considered in the context of ecological monitoring. First, volunteers were largely nonexperts and, as such, data collection were conducted at a very coarse taxonomic resolution. Consequently, it was not possible to assess faunal richness or diversity at the survey sites, and the resultant baseline against which to detect future change is somewhat limited. Second, following quality control, a significant proportion of the dataset was deemed unsuitable for inclusion in the formal analysis, due to the limited experience and/or competence of the divers or the stage of the project. Third, the use of volunteer recreational divers occasionally posed logistical issues with regards to personnel and diving operations (e.g., “no shows,” variable commitment levels, unpredictable number of divers, etc.), which would have been less of an issue within a commercial setting. Even so, CSI yielded useful ecological data because volunteers were competent and well‐trained and the project was well resourced, with adequate funding (both staff time and direct costs) to provide in‐depth training and allow for extensive survey time. Overall, the CSI project provides a useful example of how citizen science can help fill a pressing knowledge gap in our understanding of the ecological structure and health of critical marine habitats across large spatial scales.

## CONFLICT OF INTEREST

None declared.

## AUTHOR CONTRIBUTIONS

MP led the CSI project and co‐ordinated field surveys, data collection, and data management. DS led data analysis with input from GE and led manuscript preparation with input from MA. All authors contributed to manuscript development and revisions.

## Data Availability

Data are available from the Dryad Digital Repository (https://doi.org/10.5061/dryad.r06g604).
