# Supplementary material for: Spatiotemporal variability in the structure of seagrass meadows and associated macrofaunal assemblages in southwest England (UK): Using citizen science to benchmark ecological pattern
Source: Ecol Evol. 2019 Mar 4;9(7):3958–72. doi: 10.1002/ece3.5025 (PMC6467847; doi:10.1002/ece3.5025)
Supplement: Supplementary file 1 [file ECE3-9-3958-s001.docx]

**Supplementary Material**

**Spatiotemporal variability in the structure of seagrass meadows and associated macrofaunal assemblages in southwest England (UK): using citizen science to benchmark ecological pattern**

Dan A. Smale^1*^, Graham Epstein^1,2^, Mark Parry^3^, Martin J Attrill^4^

^1^Marine Biological Association of the United Kingdom, The Laboratory, Citadel Hill, Plymouth PL1 2PB, UK

^2^Ocean and Earth Science, University of Southampton, National Oceanography Centre Southampton, Waterfront Campus, European Way, Southampton SO14 3ZH, UK

^3^National Marine Aquarium, Rope Walk, Coxside, Plymouth PL4 0LF, UK

^4^School of Biological and Marine Sciences, University of Plymouth, Drake Circus, Plymouth, PL4 8AA, UK

*Corresponding author. Email: dansma@mba.ac.uk Tel: +44 (0)1752 426489

**Table S1** Overall percentage of quadrats in which each of the 9 faunal groups was present, and their overall mean abundance. Values calculated over the entire dataset.

| **Group** | **% quadrats present** | **Mean abundance (per 0.25 m^2^)** |
| --- | --- | --- |
| Molluscs | 52.0 | 3.05 |
| Worms | 53.1 | 2.20 |
| Cnidarians | 23.6 | 1.49 |
| Bryozoans | 21.0 | 1.76 |
| Fish | 20.1 | 0.32 |
| Crustaceans | 14.4 | 0.19 |
| Echinoderms | 11.8 | 0.43 |
| Ascidians | 8.0 | 0.39 |
| Sponges | 2.8 | 0.07 |

**Table S2** Post-hoc pairwise tests for seagrass presence-absence and seagrass density GLMMs (Table 2), testing for differences between locations and years. Chi-squared tests shown with Chi-squared statistic (χ^2^) and holm adjusted p-values. Significant contrasts shown in bold (ɑ<0.05).

| **Year** | **Location** | **Seagrass presence-absence** | |  | **Number of shoots (per 0.25 m^2^)** | |
| --- | --- | --- | --- | --- | --- | --- |
|  |  | χ^2^ | p |  | χ^2^ | p |
| 2016-2017 | A | 3.70 | 0.109 |  | **70.80** | **<0.001** |
| 2016-2017 | B | 13.70 | **<0.001** |  | **41.00** | **<0.001** |
| 2016-2017 | C | <0.01 | 0.972 |  | **28.86** | **<0.001** |
| 2016 | A-B | <0.01 | 1.000 |  |  |  |
| 2016 | A-C | 0.08 | 1.000 |  |  |  |
| 2016 | B-C | 0.06 | 1.000 |  |  |  |
| 2017 | A-B | 2.63 | 0.630 |  |  |  |
| 2017 | A-C | 0.07 | 1.000 |  |  |  |
| 2017 | B-C | 1.42 | 1.000 |  |  |  |

**Table S3** Post-hoc pairwise tests for total faunal abundance GLMM (Table 3), testing for differences between areas of seagrass presence-absence. Chi-squared tests shown with Chi-squared statistic (χ^2^) and holm adjusted p-values. Significant contrasts shown in bold (ɑ<0.05).

| **Year** | **Location** | **Seagrass** | **χ^2^** | **p** |  |
| --- | --- | --- | --- | --- | --- |
| 2016 | A | P-A | 43.16 | **<0.001** |  |
| 2016 | B | P-A | 5.19 | **0.039** |  |
| 2016 | C | P-A | 18.15 | **<0.001** |  |
| 2017 | A | P-A | 29.16 | **<0.001** |  |
| 2017 | B | P-A | 5.46 | **0.039** |  |
| 2017 | C | P-A | 60.57 | **<0.001** |  |

**Table S4** Post-hoc pairwise tests for total faunal abundance GLMM (Table 3), testing for differences between year and location within areas of seagrass. Chi-squared tests shown with Chi-squared statistic (χ^2^) and holm adjusted p-values. Significant contrasts shown in bold (ɑ<0.05).

| **Year** | **Location** | **χ^2^** | **p** |
| --- | --- | --- | --- |
| 2016-2017 | A | 8.67 | **0.003** |
| 2016-2017 | B | 11.55 | **0.002** |
| 2016-2017 | C | 12.07 | **0.002** |
| 2016 | A-B | 1.01 | 0.948 |
| 2016 | A-C | 2.27 | 0.789 |
| 2016 | B-C | 0.38 | 1.000 |
| 2017 | A-B | 1.77 | 0.920 |
| 2017 | A-C | 1.46 | 0.920 |
| 2017 | B-C | <0.01 | 1.000 |

**Table S5** nbGLMMs of the abundance of four dominant faunal groups, comparing quadrats containing and lacking seagrass. The effect of year and location was also considered in each model. Each coefficient is shown with the number of groups (Grps) or degrees of freedom (df), along with the associated standard deviation (Stdev), mean-squares (MS), chi-squared value (χ^2^) and p-value (p). Significant coefficients shown in bold (ɑ<0.05).

| Coefficient |  | Molluscs | | |  | Worms | | |  | Cnidarians | | |  | Bryozoans | | |
| --- | --- | --- | --- | --- | --- | --- | --- | --- | --- | --- | --- | --- | --- | --- | --- | --- |
| *Random* | Grps | Stdev | χ^2^ | p |  | Stdev | χ^2^ | p |  | Stdev | χ^2^ | p |  | Stdev | χ^2^ | p |
| Site | 19 | 0.66 | 200.89 | **<0.001** |  | 0.59 | 159.78 | **<0.001** |  | 0.92 | 174.60 | **<0.001** |  | 1.11 | 110.12 | **<0.001** |
|  |  |  |  |  |  |  |  |  |  |  |  |  |  |  |  |  |
| *Fixed* | df | MS | χ^2^ | p |  | MS | χ^2^ | p |  | MS | χ^2^ | p |  | MS | χ^2^ | p |
| Year | 1 | 82.71 | 62.02 | **<0.001** |  | 5.06 | 6.04 | **0.014** |  | 0.61 | 0.45 | 0.503 |  | 34.53 | 15.44 | **<0.001** |
| Location | 2 | 6.65 | 5.54 | 0.063 |  | 0.26 | 0.50 | 0.780 |  | 4.81 | 4.49 | 0.106 |  | 0.41 | 0.61 | 0.739 |
| Seagrass | 1 | 0.44 | 0.45 | 0.503 |  | 10.27 | 11.10 | **<0.001** |  | 39.16 | 47.66 | **<0.001** |  | 148.39 | 111.71 | **<0.001** |
| Year*Location | 2 | 23.54 | 38.28 | **<0.001** |  | 4.39 | 6.38 | **0.041** |  | 3.12 | 13.61 | **<0.001** |  | 10.56 | 17.99 | **<0.001** |
| Year*Seagrass | 1 | 0.18 | 0.04 | 0.837 |  | 8.06 | 7.24 | **0.007** |  | 4.15 | 18.86 | **<0.001** |  | 8.71 | 8.34 | **0.004** |
| Location*Seagrass | 2 | 12.61 | 22.20 | **<0.001** |  | 1.09 | 1.93 | 0.381 |  | 97.38 | 182.63 | **<0.001** |  | 0.37 | 0.29 | 0.836 |
| Year*Location*Seagrass | 2 | 16.02 | 27.58 | **<0.001** |  | 5.21 | 9.65 | **0.008** |  | 1.25 | 2.27 | 0.322 |  | 8.91 | 15.0 | **<0.001** |

**Table S6** Post-hoc pairwise tests for faunal group GLMMs (Table S5), testing for differences between areas of seagrass presence-absence. Chi-squared tests shown with Chi-squared statistic (χ^2^) and holm adjusted p-values. Significant contrasts shown in bold (ɑ<0.05).

| Year | Location | Seagrass | Molluscs | |  | Worms | |  | Cnidarians | |  | Bryozoans | |
| --- | --- | --- | --- | --- | --- | --- | --- | --- | --- | --- | --- | --- | --- |
|  |  |  | χ^2^ | p |  | χ^2^ | p |  | χ^2^ | p |  | χ^2^ | p |
| 2016 | A | P-A | 0.15 | 1.000 |  | 12.97 | **0.002** |  | 63.68 | **<0.001** |  | 44.96 | **<0.001** |
| 2016 | B | P-A | 0.28 | 1.000 |  | 3.92 | 0.191 |  | 27.77 | **<0.001** |  | 61.07 | **<0.001** |
| 2016 | C | P-A | 0.29 | 1.000 |  | 3.84 | 0.191 |  | 57.61 | **<0.001** |  | 9.38 | **0.005** |
| 2017 | A | P-A | 2.97 | 0.339 |  | 1.55 | 0.254 |  | 51.99 | **<0.001** |  | 2.86 | 0.091 |
| 2017 | B | P-A | 21.57 | **<0.001** |  | 6.24 | 0.063 |  | 0.32 | 0.572 |  | 9.90 | **0.005** |
| 2017 | C | P-A | 26.72 | **<0.001** |  | 2.33 | 0.254 |  | 26.54 | **<0.001** |  | 26.32 | **<0.001** |

**Table S7** nbGLMMs of the abundance of four dominant faunal groups, modelling the effect of the number of shoots in quadrats containing seagrass. The effect of year and location was also considered in each model. Each coefficient is shown with the number of groups (Grps) or degrees of freedom (df), along with the associated standard deviation (Stdev), mean-squares (MS), chi-squared value (χ^2^) and p-value (p). Significant coefficients shown in bold (ɑ<0.05).

| Coefficient |  | Molluscs | | |  | Worms | | |  | Cnidarians | | |  | Bryozoans | | |
| --- | --- | --- | --- | --- | --- | --- | --- | --- | --- | --- | --- | --- | --- | --- | --- | --- |
| *Random* | Grps | Stdev | χ^2^ | p |  | Stdev | χ^2^ | p |  | Stdev | χ^2^ | p |  | Stdev | χ^2^ | p |
| Site | 19 | 0.75 | 222.47 | **<0.001** |  | 0.64 | 112.14 | **<0.001** |  | 0.99 | 113.39 | **<0.001** |  | 1.25 | 95.02 | **<0.001** |
|  |  |  |  |  |  |  |  |  |  |  |  |  |  |  |  |  |
| *Fixed* | df | MS | χ^2^ | p |  | MS | χ^2^ | p |  | MS | χ^2^ | p |  | MS | χ^2^ | p |
| Year | 1 | 50.71 | 31.67 | **<0.001** |  | 0.19 | 0.25 | 0.615 |  | 3.11 | <0.01 | 0.961 |  | 30.41 | 51.42 | **<0.001** |
| Location | 2 | 1.28 | 2.07 | 0.354 |  | 0.17 | 0.31 | 0.856 |  | 8.86 | 12.51 | **0.002** |  | 0.26 | 0.14 | 0.931 |
| Seagrass | 1 | 4.35 | 3.24 | 0.072 |  | 0.95 | 1.63 | 0.202 |  | 47.46 | 35.34 | **<0.001** |  | 120.13 | 98.75 | **<0.001** |
| Year*Location | 2 | 18.02 | 25.99 | **<0.001** |  | 7.51 | 10.93 | **0.004** |  | 7.41 | 14.68 | **<0.001** |  | 4.52 | 8.83 | **0.012** |
| Year*Seagrass | 1 | 0.53 | 0.615 | 0.433 |  | 0.07 | 0.02 | 0.880 |  | 0.65 | 0.29 | 0.592 |  | 4.08 | 3.97 | **0.046** |
| Location*Seagrass | 2 | 1.60 | 3.92 | 0.141 |  | 0.23 | 0.57 | 0.752 |  | 11.32 | 21.01 | **<0.001** |  | 4.29 | 6.03 | **0.049** |
| Year*Location*Seagrass | 2 | 2.60 | 4.30 | 0.117 |  | 5.60 | 11.08 | **0.004** |  | 3.39 | 6.91 | 0.032 |  | 4.14 | 6.28 | **0.043** |

**Table S8** Post-hoc pairwise PERMANOVA for multivariate faunal assemblage structure (Table 4), testing for differences between areas of seagrass presence-absence. Pairwise tests shown with associated t-value (t) and p-values (p). Significant contrasts shown in bold (ɑ<0.05).

| Year | Location | Seagrass | t | p |  |
| --- | --- | --- | --- | --- | --- |
| 2016 | A | P-A | 3.77 | **<0.001** |  |
| 2016 | B | P-A | 3.24 | **<0.001** |  |
| 2016 | C | P-A | 4.57 | **<0.001** |  |
| 2017 | A | P-A | 3.10 | **<0.001** |  |
| 2017 | B | P-A | 2.02 | **0.011** |  |
| 2017 | C | P-A | 5.17 | **<0.001** |  |

**Table S9** Post-hoc pairwise PERMANOVA for multivariate faunal assemblage structure (Table 4), testing for differences between years and locations in areas of seagrass. Pairwise tests shown with associated t-value (t) and p-values (p). Significant contrasts shown in bold (ɑ<0.05).

| Year | Location | t | p |
| --- | --- | --- | --- |
| 2016-2017 | A | 1.45 | 0.120 |
| 2016-2017 | B | 1.27 | 0.216 |
| 2016-2017 | C | 0.70 | 0.564 |
| 2016 | A-B | 1.49 | **0.040** |
| 2016 | A-C | 1.26 | 0.162 |
| 2016 | B-C | 1.13 | 0.295 |
| 2017 | A-B | 0.76 | 0.728 |
| 2017 | A-C | 0.81 | 0.674 |
| 2017 | B-C | 0.59 | 0.921 |

**Table S10** Post-hoc pairwise PERMANOVA for multivariate faunal assemblage structure (Table 4), testing for differences between seagrass density categories in areas of seagrass, for each location and year separately. Pairwise tests shown with associated t-value (t) and p-values (p). Significant contrasts shown in bold (ɑ<0.05).

|  |  | 2016 | |  | 2017 | |
| --- | --- | --- | --- | --- | --- | --- |
| Density Cat | Location | t | p |  | t | p |
| E-C | A | 1.87 | **0.009** |  | 1.91 | **0.012** |
| E-D | A | 1.44 | 0.099 |  | 1.56 | 0.056 |
| E-B | A | 2.04 | **0.004** |  | 2.03 | **0.006** |
| C-D | A | 1.80 | **0.015** |  | 1.21 | 0.225 |
| C-B | A | 1.67 | **0.024** |  | 1.23 | 0.231 |
| D-B | A | 2.46 | **<0.001** |  | 1.06 | 0.370 |
| E-C | B | 1.64 | 0.052 |  | 1.44 | 0.116 |
| E-D | B | 1.32 | 0.174 |  | 1.21 | 0.235 |
| E-B | B | 1.71 | **0.038** |  | 1.80 | **0.023** |
| C-D | B | 1.37 | 0.131 |  | 1.33 | 0.154 |
| C-B | B | 1.27 | 0.213 |  | 0.37 | 0.878 |
| D-B | B | 1.76 | **0.022** |  | 1.44 | 0.099 |
| E-C | C | 1.08 | 0.366 |  | 1.06 | 0.379 |
| E-D | C | 1.18 | 0.282 |  | 0.88 | 0.501 |
| E-B | C | 1.48 | 0.146 |  | 0.93 | 0.473 |
| C-D | C | 0.48 | 0.848 |  | 0.86 | 0.557 |
| C-B | C | 1.24 | 0.219 |  | 1.13 | 0.305 |
| D-B | C | 1.28 | 0.212 |  | 1.16 | 0.293 |

**Fig S1.** Mean abundance (± se) of four dominant groups in each location and year, for quadrats containing (darker bars to the right) and lacking (lighter bars to the left) seagrass.


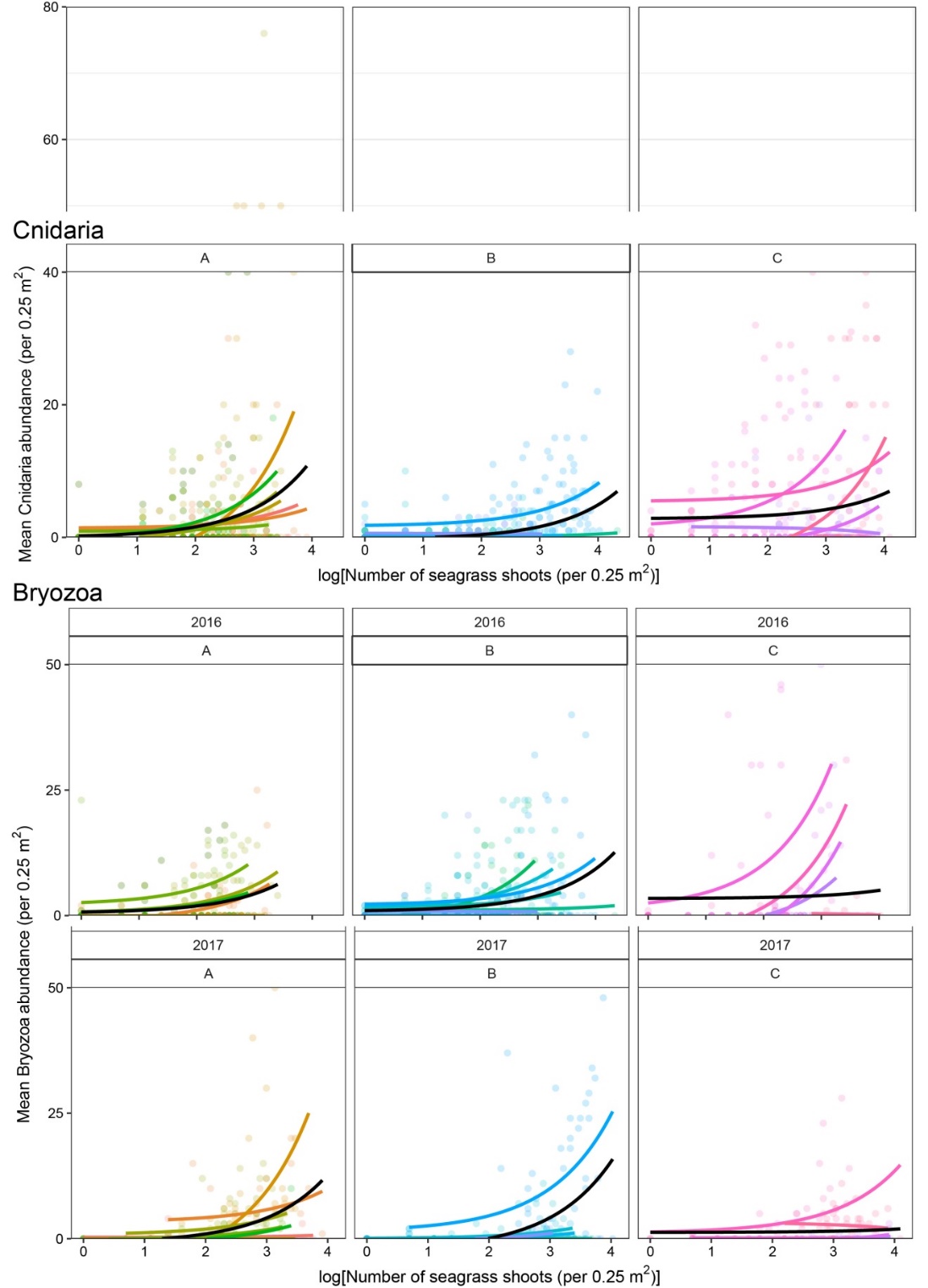


**Fig S2.** Scatter plots indicating the effect of seagrass density on mean Cnidaria and Bryozoa abundance within each location and year where relevant to global nbGLMMs. Each point represents a single quadrat, with colours separating individual sites. Coloured lines are exponential smoothing functions for each site; while black lines are exponential smoothing functions across sites.

**Fig S3** Threshold metric multi-dimensional scaling (tmMDS) plots of bootstrapped average fauna community data from quadrats containing seagrass. tmMDS grouped to location and year. Bootstrapping and tmMDS based on Bray-Curtis distance matrices constructed from 4^th^ root transformed data.
